# Edge advances in nanodrug therapies for osteoarthritis treatment

**DOI:** 10.3389/fphar.2024.1402825

**Published:** 2024-10-30

**Authors:** Jinfeng Liao, Qingjia Gu, Zheng Liu, Hailian Wang, Xian Yang, Rongkai Yan, Xiaofeng Zhang, Siyuan Song, Lebin Wen, Yi Wang

**Affiliations:** ^1^ Department of Dermatology, Sichuan Academy of Medical Science and Sichuan Provincial People’s Hospital, Chengdu, Sichuan, China; ^2^ Department of Neuroscience, Baylor College of Medicine, Houston, TX, United States; ^3^ Department of ENT, Sichuan Academy of Medical Science and Sichuan Provincial People’s Hospital, Chengdu, Sichuan, China; ^4^ Clinical Immunology Translational Medicine Key Laboratory of Sichuan Province, Center of Organ Transplantation, Sichuan Academy of Medical Science and Sichuan Provincial People’s Hospital, Chengdu, Sichuan, China; ^5^ Department of Critical Care Medicine, Sichuan Academy of Medical Sciences and Sichuan Provincial People’s Hospital, School of Medicine, University of Electronic Science and Technology of China, Chengdu, China; ^6^ Department of Radiology, Ohio state university, Columbus, OH, United States; ^7^ Greenwich Hospital, Yale New Haven Health, Greenwich, CT, United States; ^8^ Department of Thyroid, Sichuan Second Hospital of TCM, Chengdu, China

**Keywords:** osteoarthritis, nanodrug, immunological responses, mesenchymal stem cells, signaling pathway

## Abstract

As global population and lifestyles change, osteoarthritis (OA) is becoming a major healthcare challenge world. OA, a chronic condition characterized by inflammatory and degeneration, often present with joint pain and can lead to irreversible disability. While there is currently no cure for OA, it is commonly managed using nonsteroidal anti-inflammatory drugs (NSAIDs), glucocorticoids, and glucosamine. Although these treatments can alleviate symptoms, it is difficult to effectively deliver and sustain therapeutic agents within joints. The emergence of nanotechnology, particularly in form of smart nanomedicine, has introduced innovative therapeutic approaches for OA treatment. Nanotherapeutic strategies offer promising advantages, including more precise targeting of affected areas, prolonged therapeutic effects, enhanced bioavailability, and reduced systemic toxicity compared to traditional treatments. While nanoparticles show potential as a viable delivery system for OA therapies based on encouraging lab-based and clinical trials results, there remails a considerable gap between current research and clinical application. This review highlights recent advances in nanotherapy for OA and explore future pathways to refine and optimize OA treatments strategies.

## 1 Introduction

Osteoarthritis (OA) is a prevalent condition, affecting over 500 million people worldwide, which is equates to approximately 7% of the world’s population (Duan et al., 2023). From 1990 to 2019, the prevalence of OA increased by 48% (Hunter et al., 2020), underscoring its growing impact. Research increasingly indicates that factors such as obesity, aging, poor dietary habits, and hypertension significantly contribute to the progression of OA (Colletti and Cicero, 2021). The primary objectives in managing OA are pain relief, reduce of joint inflammation, enhancing joint function, and minimizing overall disability (Filardo et al., 2015; De Faro Silva et al., 2022). Standard treatment typically involves intra-articular drug injections and surgical interventions (Ebert et al., 2013); however, these approaches often fall short in efficacy. Surgical treatments, in particular, are complex and require prolonged recovery periods (Conaghan et al., 2019). While commonly used clinical treatments like NSAIDs, glucocorticoids, and glycosaminoglycan can provide symptomatic relief, but do little to halt the progression of OA (Katz et al., 2021), highlighting the urgent need for more effective treatments.

In recent decades, significant progress has been made in the field of nanomedicine for treating various diseases (Janowicz et al., 2022; Zhang Z. et al., 2022; Han and Huang, 2023). In the context of OA, researchers are working on developing nanocarrier systems to utilize nanoparticles to extend the duration of drug efficacy, allowing for a gradual release of the therapeutic agents and improving their penetration into chondrocytes or synovial cells (Mitchell et al., 2021; Dilliard et al., 2021; Boehnke et al., 2022). Despite these promising advancements, no nanotherapeutic drugs for OA have yet received clinical approval. This review aims to explore recent developments in nanomedicine for OA treatment, providing a comprehensive overview of its properties, potential benefits, and the challenges that must be addressed for clinical application.

## 2 Pathogenesis of osteoarthritis

Osteoarthritis (OA) is characterized by a complex interplay of pathological changes within affected joints, including the deterioration of articular cartilage (Krishnan and Grodzinsky, 2018), inflammation of the synovial membrane (Hu et al., 2019), remodeling of the subchondral bone (Zhu et al., 2021), and the development of osteophytes (McCulloch et al., 2017). The widely accepted theory is that OA arises from an imbalance between degradation and repair of cartilage (Guilak et al., 2018). Articular cartilage, which is crucial for smooth joint movement, consists primarily of chondrocytes embedded in an extracellular matrix (ECM) rich in type II collagen and proteoglycans (Zou et al., 2021). Under normal conditions, chondrocytes maintain cartilage integrity by synthesizing type II collagen and proteoglycans while also regulating the activity of enzymes such as matrix metalloproteinases (MMPs). These enzymes are responsible for maintaining a balance between the breakdown (catabolism) and synthesis (anabolism) of cartilage components (Guo et al., 2018; Li T. et al., 2022).

Various factors, including mechanical stress, metabolic changes, aging, and inflammation, contribute to the progression of OA. Mechanical stress, such as that caused by joint overuse or injury, can lead to the release of damage-associated molecular patterns (DAMPs) from damaged tissues, which activate pattern recognition receptors (PRRs) on immune cells line macrophages (Li and Wu, 2021). This, in turn, stimulates the production of pro-inflammatory cytokines, including Tumor Necrosis Factor-alpha (TNF-α) and Interleukin-1β (IL-1β) (Molnar et al., 2021). Obesity exacerbates this process through chronic low-grade inflammation, known as “metaflammation”, where expanded adipose tissue expansion leads to increased macrophage infiltration pro-inflammatory M1 phenotype, characterized by high levels of TNF-α and IL-1β production (Li et al., 2023). Aging further contributes to OA through “inflammaging,” a state of chronic, low-grade inflammation driven by the accumulation of senescent cells, oxidative stress, and altered immune function, all of which contribute to increased production of inflammatory (Chow and Chin, 2020; Wojdasiewicz et al., 2014; Liu et al., 2022a).

Inflammatory cytokines have a profound effect on chondrocytes, leading to phenotypic changes that disrupt cartilage homeostasis. These cytokines suppress the production of type II collagen and proteoglycans in chondrocytes by activating pathways like Mitogen-Activated Protein Kinase (MAPK) and Nuclear Factor-kappa B (NF-κB), particularly through the influence of mediator like prostaglandin E2 (PGE-2), Nitric Oxide (NO), and cyclooxygenase-2 (COX-2) (Ma et al., 2016; Teng et al., 2023; Chen et al., 2018; Yao et al., 2022; Lu R. et al., 2023). This shift increases the production of specific MMPs (e.g., MMP1, MMP3, MMP9, MMP13) while reducing the synthesis of collagen and proteoglycans (Ling et al., 2021; Tian et al., 2021), which collectively contribute to the breakdown of the ECM.

**FIGURE 1:**
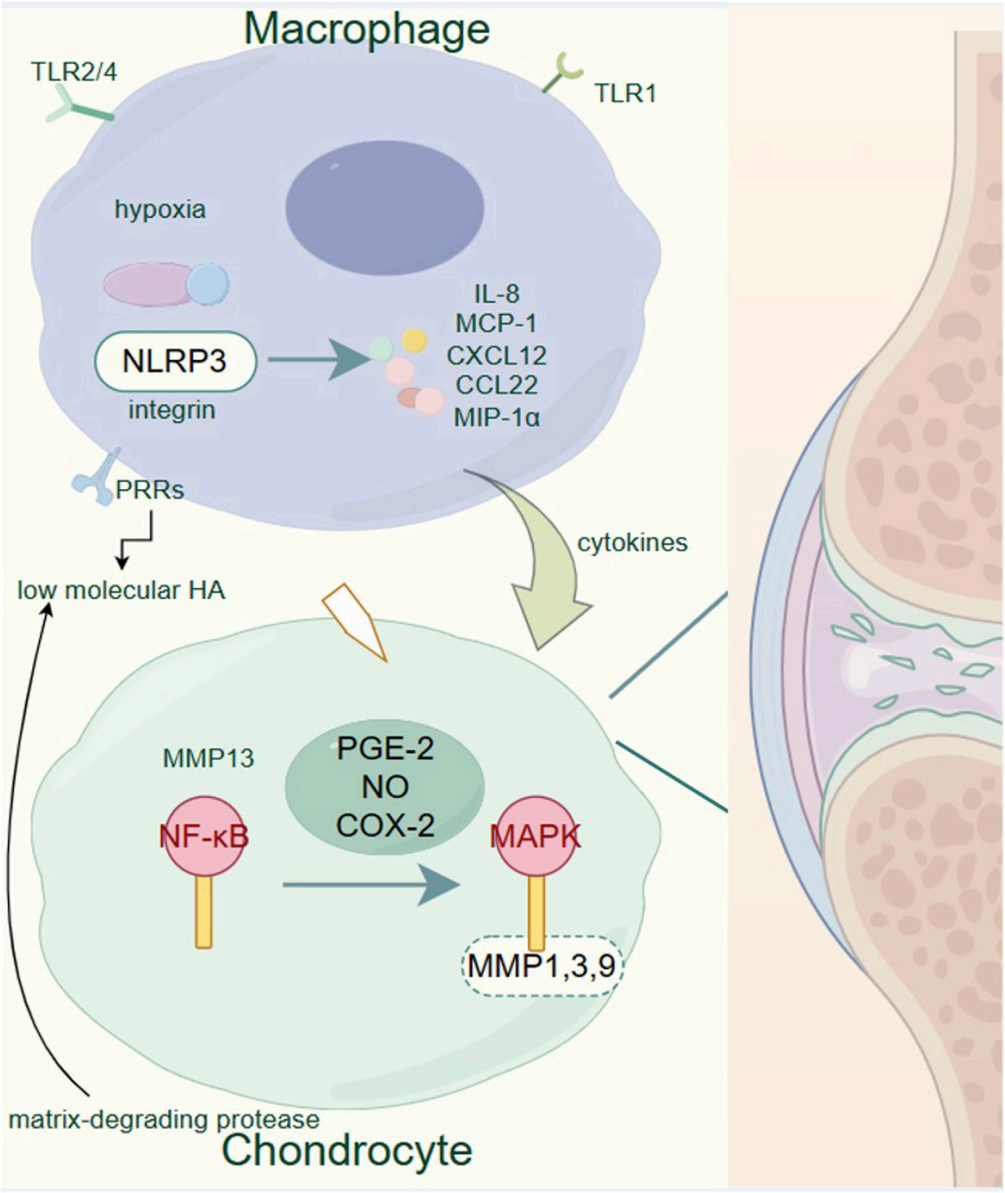
Pathogenesis of osteoarthritis. Within the OA-affected joints, macrophages play a critical role by secreting pro-inflammatory cytokines and chemokines such as IL-8, MCP-1, CXCL12, CCL22, and MIP-1α in response to various stimuli including hypoxia and low molecular HA. These inflammatory mediators contribute to the activation of chondrocytes. Activated chondrocytes express elevated levels of matrix-degrading proteases, particularly MMP1, 3, 9, and 13, via the NF-κB and MAPK signaling pathways, induced by pro-inflammatory factors like PGE-2, NO, and COX-2. This upregulation of proteases leads to the degradation of the extracellular matrix, a hallmark of OA progression. The figure underscores the importance of the NF-κB and MAPK pathways in the catabolic processes of cartilage degradation, highlighting potential targets for therapeutic intervention to mitigate the progression of OA.

As ECM breaks down, it releases various molecular fragments that act as DAMPs, further activating PRRs on macrophages and chondrocytes (Son et al., 2020; Lambert et al., 2021; Foell et al., 2007; Rahmati et al., 2016; Hügle et al., 2022). This perpetuates the inflammatory cycle, enhancing the activation of NF-κB and MAPK pathways, and accelerating cartilage degradation (Moon et al., 2018; Liao et al., 2020; Xu et al., 2022; Boehme and Rolauffs, 2018). Additionally, hypoxia within the joint microenvironment exacerbates inflammation, activating the NLRP3 inflammasome in macrophages and leading to the release of pro-inflammatory cytokines such as IL-8, MCP-1, CXCL12, CCL22, and MIP-1α (Quero et al., 2020; Raghu et al., 2017; Kuang et al., 2020; Ren et al., 2021; Hou et al., 2020; Zhao et al., 2020). These cytokines contribute to the recruitment and activation of additional immune cells, further perpetuating the inflammatory cycle (Nelson et al., 2011) (Figure 1).

As OA progresses, cartilage degeneration extends into the calcified layer, promoting pathological changes in the subchondral bone, including the formation of osteophytes around the joint periphery (Hu et al., 2021; Roelofs et al., 2020). Addressing these inflammatory pathways and restoring the balance between cartilage degradation and synthesis are critical strategies in preventing and treating OA.

## 3 Nanomedicines to reduce inflammation of synovial and articular cartilage

Current clinical approaches to OA treatment are categorized into three main types: non-pharmacologic management, pharmacologic management, and surgical interventions (Emami et al., 2018; Evans et al., 2014). Non-pharmacologic methods, such as exercise and physical therapy, are often employed to alleviate symptoms. However, there strategies primarily address the symptoms rather than the underlying pathology of OA, and in some cases, improper application may exacerbate the condition (Lin et al., 2023; Furtado et al., 2022). Surgical interventions, such as arthroscopic surgery or joint replacement, provide more direct solution but are associated with high costs, invasiveness, and substantial risks, particularly in elderly patients or those with comorbidities (Skou et al., 2015).

Pharmacologic treatments, although less invasive than surgery, also present significant limitations. Commonly used drugs such as nonsteroidal anti-inflammatory drugs (NSAIDs), glucocorticoids (GCs), glycosaminoglycans (GAGs), opioid analgesics, steroids, and hyaluronic acid (HA), can be administered via various routes, including oral, intravenous, intra-articular, and transdermal methods (McGuckin et al., 2022; Oo et al., 2021). Despite their widespread use, these pharmacologic approaches are hindered by issues such as limited local drug concentration in the joints, rapid drug clearance from synovial fluid, and systemic side effects, including gastrointestinal, renal, and cardiovascular complications, particularly with long-term use (Evans et al., 2014; Togo et al., 2022; Cooper et al., 2019).

Nanotechnology has emerged as a promising avenue for overcoming the limitations of traditional OA therapies. Nanoparticles, when utilized as drug carriers, have the unique ability to selectively accumulate in affected joints, minimizing systemic exposure and maximizing local therapeutic effects (Huang et al., 2022; Li X. et al., 2021). These carriers can also stabilize encapsulated drugs, enabling controlled and sustained release, which prolongs drug retention time and reduces site-specific toxicity (Huang et al., 2022). Moreover, nanomaterials can be engineered as stimulus–responsive drug delivery systems, triggered by external stimuli such as temperature, magnetic fields, and electric fields (Said et al., 2019). This targeted and on-demand drug delivery system enhances therapeutic efficacy while mitigating the risk of side effects.

Nanomedicine in OA treatment involves the delivery of various therapeutic agents, including small-molecule drugs, nucleic acids, and peptides/proteins, via nanomaterials designed to inhibit OA progression. By offering more precise, sustained, and responsive drug delivery, nanomedicine holds significant promise for improving OA management, addressing both symptoms and underlying pathologies, and overcoming the limitations of traditional therapies (Dou et al., 2020; Kumar et al., 2019; Xu et al., 2023).

### 3.1 Combination of nanotechnology with anti-inflammatory drugs

**FIGURE 2:**
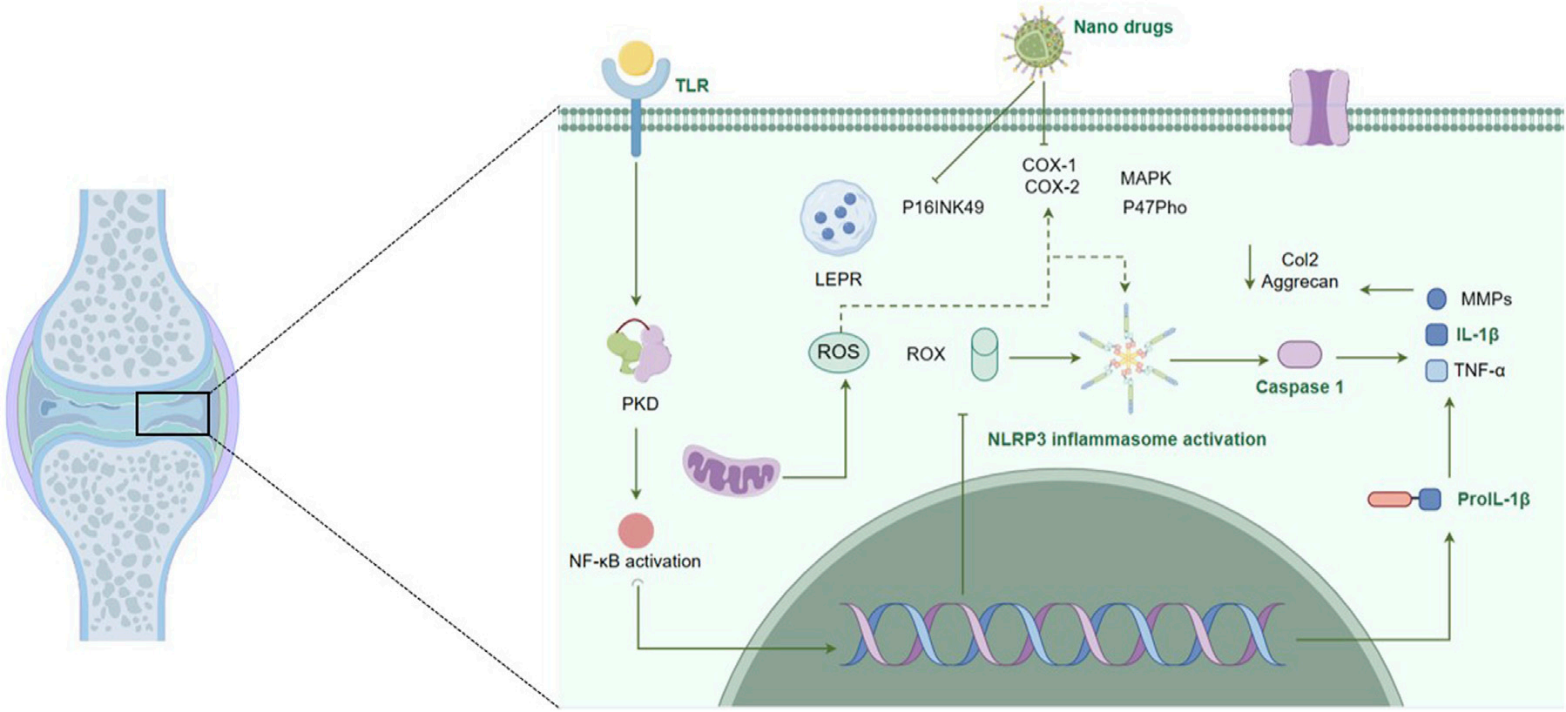
Nanomedicines and their immune regulatory role in osteoarthritis. This figure depicts the multifaceted mechanisms of action of nanomedicines in the regulation of immune responses within the pathophysiological context of osteoarthritis. It illustrates the targeted delivery and sustained release of nanoformulated drugs, highlighting their interactions with cellular and molecular components are integral to the inflammatory process and consequent cartilage degradation in osteoarthritis. The figure underscores the potential of nanomedicines to reduce inflammation by inhibiting these pathways and to enhance the bioavailability and efficacy of therapeutic agents, offering a strategic approach in osteoarthritis treatment.

OA is a multifactorial disease characterized by the progressive degradation of joint cartilage and the inflammation of synovial membranes (Scanzello and Goldring, 2012). Inflammation is a key driver of OA progression, where inflammatory cytokines, such as TNF-α and IL-1β, play a central role. Traditional pharmacological interventions like NSAIDs, glucocorticoids (GCs), steroids, and Glycosaminoglycans (GAGs), aim to alleviate symptoms by suppressing inflammation (Strokotova and Grigorieva, 2022; Magni et al., 2021; Nunes et al., 2021). However, these treatments often have limited efficacy and are associated with significant systemic side effects. The development of nanomedicines offers a promising alternative. Some nanoparticles are designed to target the inflamed synovium and cartilage directly. By encapsulating anti-inflammatory agents within these nanovesicles, these nanomedicines can achieve sustained drug release and higher local drug concentrations within the joints (Wang Y. et al., 2022; Wen et al., 2023). This targeted approach not only enhances the therapeutic potential of existing drugs, but also minimized the adverse effects. Another strategy explored the use of metallic nanoparticles. Some metallic particles can directly act on biological molecules, they can either act as nanoenzyme to reduce oxidative stress, or act through inhibition of the NF-κB signaling pathway or activation of NLRP3 inflammasome (Li R. et al., 2022; Luo et al., 2021; Ma, 2023) (Figure 2). In the following sections, we will explore the application of nanomedicines for reducing inflammation in OA, focusing on the mechanisms by which these advanced therapies can improve clinical outcomes by targeting the synovial and articular cartilage.

#### 3.1.1 Nonsteroidal anti-inflammatory drugs (NSAIDs)

NSAIDs such as ibuprofen, flurbiprofen, diclofenac, celecoxib, indomethacin, meloxicam, piroxicam, and naproxen, are among the most widely prescribed medications for alleviating symptoms in OA patients. Their primary mode of action involves robust inhibition of cyclooxygenase (COX) enzymes, particularly COX-1 and COX-2 at sites of inflammation, which in turn suppresses the biosynthesis of prostaglandins (PGs) (Ahmadi et al., 2022; Eisenstein et al., 2022; Stiller and Hjemdahl, 2022; Busa et al., 2022). PGs play a key role in sensitizing pain pathways by interacting with TRPV1, Nav1.8 and other ion channels to reduce their threshold, leading to heightened pain perception, amplifying pain perception (Magni et al., 2021; Zhu et al., 2020).

Besides their primary COX-inhibitory action, NSAIDs also modulate various other inflammatory pathways in OA, they reduce the production of pro-inflammatory cytokines and leukotrienes, which are central to joint inflammation and tissue degradation (Alvarez-Soria et al., 2006; Maślanka and Jaroszewski, 2013; Hassan and Ghobara, 2016; Li et al., 2018; Nagy et al., 2017). Furthermore, NSAIDs exert immunomodulatory effects by inhibiting the activation and migration of immune cells (Molnar et al., 2021; Li Z. et al., 2021). This broad spectrum, of anti-inflammatory actions make NSAIDs effective in managing the symptoms of OA. However, long-term use of NSAIDs is associated with several adverse effects, including gastrointestinal (GI) distress, peptic and duodenal ulcers, small bowl erosion, colitis, acute renal failure, hypertension, chronic kidney disease, heart failure, myocardial infarction, stroke, seizures, and delayed wound healing (Sohail et al., 2023; Chevalier et al., 2009). These risks have driven research towards improving NSAID delivery methods to enhance their therapeutic index while minimizing systemic side effects.

Nanotechnology has significantly advanced the field of drug delivery, offering innovative solutions to overcome the limitation associated with traditional drug delivery. Encapsulating NSAIDs within nanoscale carriers, provides several advantages, including protection from premature degradation, targeted drug delivery, and controlled release (Ashfaq et al., 2023). These nanocarriers typically range from 10–200 nm in size, typical nanocarriers that are used to encapsulating NSAIDs are liposomes, polymeric nanoparticles, and solid lipid nanoparticles (SLNs) (Badri et al., 2016; Pontes et al., 2022).

Liposomes are composed of lipid bilayers, which can encapsulate both hydrophilic and hydrophobic NSAIDs (Lee, 2020). Liposomes can be engineered to release the drug in response to specific stimuli, such as changes in pH or temperature, making them effective for localized delivery in inflamed joints (Liu P. et al., 2022). This stimulus-responsive release mechanism is particular effective for localized drug delivery with inflamed joints thereby reducing systemic exposure and associated side effects.

Polymeric nanoparticles are made from biocompatible and biodegradable polymers, these nanoparticles can encapsulate NSAIDs, ensuring a sustained release over time (Baek et al., 2017). This controlled release minimizes the need for frequent dosing and reduces systemic side effects.

SLNs are composed of solid lipids, which remain solid at body temperature. The surface of SLNs can be further coated with enteric polymers that are resistant to acidic environments, by using the combination of solid lipids and enteric coatings, SLNs are designed to bypass the stomach without releasing their contents (Pandey et al., 2021). The SLNs then release the NSAID once they reach the more neutral pH of the intestines or are absorbed into the bloodstream (Mancini et al., 2021). Once the SLNs reach the joint environment, their stability is influenced by the specific modifications made to the nanoparticle (Hsu et al., 2023). For example, SLNs can be designed to degrade in response to enzymes that are overexpressed in the inflamed joint, such as matrix metalloproteinases (MMPs). When these enzymes come into contact with the SLNs, they break down the solid lipid matrix, releasing the encapsulated NSAID directly into the inflamed tissue (Chuang et al., 2018). This coating dissolves only in the more neutral pH of the small intestine, allowing the SLNs to remain intact until they are absorbed into the bloodstream. SLNs offer a stable platform for NSAID delivery, with enhanced bioavailability and reduced gastrointestinal side effects due to localized drug release (Singh et al., 2019).

In comparison to SLNs or liposomes, inorganic nanoparticles, such as silica (SiO_2_), gold (AuNPs), iron oxide (Fe_2_O_3_ or Fe_3_O_4_), or cerium oxide (CeO_2_), provide a unique advantage in delivering NSAIDs for OA treatment because of their structural robustness, precise targeting capabilities, and high stability in various physiological environments (Corsi et al., 2023; Pourmadadi et al., 2022; He et al., 2021; Kalashnikova et al., 2020). Inorganic nanoparticles are inherently stable under a wide range of pH conditions, including the acidic environment of the stomach, making them highly effective for drug delivery, including NSAID encapsulation. Their robust structure prevents the encapsulated NSAID from being exposed to gastric acid, thereby protecting it from premature degradation (Hsu et al., 2023). The surface of inorganic nanoparticles can be modified with targeting ligands, such as antibodies, peptides, or small molecules that recognize and bind to specific receptors overexpressed in inflamed joints. For example,: folate receptors, which are often overexpressed in inflamed tissues, can be targeted by modifying the surface of inorganic nanoparticles with folate molecules. This targeting mechanism can lead to increased drug accumulation in the inflamed joint, improving therapeutic efficacy while reducing systemic side effects (Yu et al., 2010; Sanità et al., 2020; Huang et al., 2024). In the context of OA treatment, inorganic nanoparticles can enhance the bioavailability and therapeutic action of NSAIDs by providing protection against premature degradation, enabling controlled release, and improving tissue targeting (Yi et al., 2024).

#### 3.1.2 Glycosaminoglycans (GAGs)

Glycosaminoglycans (GAGs), such as hyaluronic acid (HA), chondroitin sulfate, and keratan sulfate, are essential components of the extracellular matrix in cartilage. They help maintain the structural integrity and mechanical function of cartilage by providing lubrication and shock absorption in joints. HA is the most commonly used GAG in the treatment of OA (Altman et al., 2015; Testa et al., 2021; Jargin, 2012). HA products vary in molecular weight and cross-linking, which influences their viscosity and duration of action within the joint. These formulations are either derived from bacterial fermentation or extracted from rooster combs and must undergo purification to remove impurities (Serra et al., 2023).

HA is administered via intra-articular injection to restore the viscoelastic properties of synovial fluid, reducing pain and improving joint mobility (Legré-Boyer, 2015; Chavda et al., 2022). Beyond its lubricating function, HA also modulates the inflammatory response by inhibiting the activity of pro-inflammatory cytokines and enzymes, such as matrix metalloproteinases (MMPs), which degrade cartilage (Marinho et al., 2021). Furthermore, HA can inhibit the activation of the NF-κB pathway, thereby reducing the expression of inflammatory mediators like COX-2 and MMPs (Marinho et al., 2021). It may also influence the MAPK signaling pathway, affecting cell survival and inflammation (Chen et al., 2019).

Since HA is typically applied directly into the affected joint, nanoparticle technologies for HA-based treatments aim to overcome different challenges than those associated with NSAIDs. While NSAIDs nanoparticles formulations target tissue-specific delivery to inflamed joints, HA-based treatments focus on extending the therapeutic effects, enhancing stability, and improving cartilage penetration. One key limitation of HA is its rapid degradation due to enzymatic activity and its large molecular size, which restricts penetration into deeper cartilage layers (Gan et al., 2024). To address these issues, various nanoparticle technologies have been explored. For instance, poly (lactic-co-glycolic acid) (PLGA) nanoparticles can encapsulate HA, protecting it from enzymatic degradation and enabling sustained release over time. Additionally, PLGA nanoparticles can also be modified with polyethylene glycol (PEG) to increase circulation time and reduce immune clearance (Pontes et al., 2022; Zerrillo et al., 2022; Householder et al., 2023). Natural polymers such as chitosan, alginate, and cellulose derivatives show therapeutic potential for intra-articular drug delivery. Chitosan is another promising natural polymer for nanoparticle formation with HA. Its mucoadhesive properties help retain the nanoparticles in the joint, and its positive charge facilitates interaction with the negatively charged cartilage, improving penetration (Pontes et al., 2022). Other nanoparticle systems, such as hollow mesoporous silica nanoparticles (HMSNs) and liposomes can also be used to encapsulate HA. HMSNs offer a porous surface for control release, while liposomes protect HA from enzymatic degradation and allow for sustained delivery (Wen et al., 2023; Teixeira et al., 2022).

Overall, the application of nanoparticle technologies in HA-based OA treatments focuses on prolonging the therapeutic effects of HA, enhancing its stability, and improving its penetration into cartilage tissue, rather than focusing on tissue-specific delivery as is the case with NSAIDs.

#### 3.1.3 Natural products from medicinal plants

Natural plant-derived medicines have gained significant attention in the treatment of OA due to their potential to offer anti-inflammatory, analgesic, and cartilage-protective effects with fewer side effects compared to conventional pharmaceuticals (Fang et al., 2024; Akhileshwar Jha et al., 2024; Mu et al., 2022). The significance of plant-based therapies in OA treatment is seen from multiple perspectives, including their historical use in traditional medicine, increasing scientific validation, and the growing interest in integrative approaches to managing chronic diseases like OA (Kuang et al., 2023). However, many plant-derived compounds face challenges such as poor bioavailability, rapid metabolism, and insufficient tissue targeting. Nanoparticle technologies present a promising solution by enhancing the delivery, bioavailability, and therapeutic efficacy of these compounds, improving their potential in OA treatment. While clinical studies on nanoparticles for OA are still in early stages, preclinical data suggest that multiple natural products-loaded nanoparticles could represent a novel and effective approach for mitigating OA symptoms and slowing disease progression (Fang et al., 2024; Cao et al., 2022).

Cannabidiol (CBD), a non-psychoactive component of *Cannabis sativa*, has been recognized for its anti-inflammatory and analgesic properties (Lowin et al., 2020). It interacts primarily with the endocannabinoid system (ECS), specifically CB1 and CB2 receptors, which play roles in modulating pain, inflammation, and immune responses (Fine and Rosenfeld, 2013; Vučković et al., 2018). CBD reduces pro-inflammatory cytokine production, inhibits immune cell, and mitigates oxidative stress, all contributing to joint protection in OA. However, due to its lipophilic nature and poor bioavailability, traditional oral administration of CBD can be inefficient (Bryk and Starowicz, 2021). Nanotechnology can enhance CBD’s delivery by encapsulating it in lipid-based nanoparticles similar as encapsulating NSAIDs. Liposomes, polymeric nanoparticles, or SLNs, have been employed to improve CBD’s solubility, bioavailability, and stability (Assadpour et al., 2023; Alcantara et al., 2024). Nanoparticles, especially those functionalized with targeting ligands, can direct CBD to inflamed joints, thereby enhancing its therapeutic potential while minimizing systemic side effects. For instance, CBD-loaded PLGA nanoparticles have been shown to enhance bioavailability, allowing for more effective delivery to target tissues, by reducing inflammation and improving the therapeutic outcomes associated with OA (Jin et al., 2023).

##### 3.1.3.1 Curcumin (Curcuma domestica)

Curcumin, a polyphenol derived from turmeric (*Curcuma longa*), is well-recognized for its anti-inflammatory properties (Salehi et al., 2019), particularly its ability to inhibit the NF-κB pathway, which is central to inflammation in OA (Buhrmann et al., 2021). Additionally, curcumin scavenges free radicals, reducing oxidative stress in affected joints. However, curcumin’s low solubility in water and rapid metabolism limits its clinical use (Crivelli et al., 2019; Guan et al., 2022). SLNs can enhance the oral bioavailability of curcumin by protecting it from the acidic environment of the stomach and enabling sustained release (Ban et al., 2020). It is also reported that the curcumin can be dissolved with mPEG (5kD)-PCL(2kD) polymer to produce the curcumin-loaded polymeric micelles, which has a 74.8 ± 8.68 days nm (Gupta et al., 2020; Kang et al., 2020). This approach can improve curcumin’s solubility and enhance its systemic circulation time, allowing for more effective delivery to the joints (Kang et al., 2020).

##### 3.1.3.2 Boswellia serrata (boswellic acid)

Boswellic acid, from *Boswellia serrata* (Indian frankincense), inhibits the 5-lipoxygenase enzyme, reducing leukotriene synthesis, which is involved in inflammation and pain (Börner et al., 2021; Gomaa et al., 2021). It also protects cartilage by reducing the degradation caused by MMPs (Shin et al., 2022). Due to its hydrophobic nature and poor gastrointestinal absorption, nanoparticle systems such as nanoemulsion and cyclodextrin inclusion complex have been developed to improve the bioavailability of boswellic acid (Ting et al., 2018). Different from nanocapsules where the drug is confined to a cavity surrounded by a polymeric membrane and are typically 10–500 nm in size, nanoemulsions are colloidal dispersions where two immiscible liquids, typically oil and water, are stabilized by surfactants. The droplets in nanoemulsions are usually in the range of 20–200 nm, with the drug is dissolved in the dispersed phase (Borrajo et al., 2024). Instead of protecting the drug from the harsh acidic environment of the stomach and the enzymatic activity in the intestine, nanoemulsions are designed to enhance the digestion of encapsulated lipophilic compounds by allowing them to be more easily absorbed through the intestinal lining. They can enhance solubility and bioactivity of boswellic acids in the gastrointestinal tract (Choi and McClements, 2020; Han et al., 2022). The small droplet size and large surface area of nanoemulsions enable better absorption and distribution of the boswellic acids, enhancing their therapeutic effects. Cyclodextrins improve the solubility and stability of hydrophobic drugs by forming inclusion complexes where the drug is hosted inside the hydrophobic cavity of the cyclodextrin molecule. Cyclodextrin complexes can help the boswellic acids dissolve more easily in the aqueous environment of the GI tract, improving its bioavailability (Sarabia-Vallejo et al., 2023). Similarly, cyclodextrin complexations do not protect boswellic acids from the acidic environment like nanocapsules but rather enhance the drug’s solubility and absorption in the intestines (Tambe et al., 2018).

##### 3.1.3.3 Quercetin

Quercetin, a flavonoid found in various fruits and vegetables, exhibits anti-inflammatory effects by inhibiting the production of pro-inflammatory cytokines and oxidative stress, as well as stabilization lysosomal membranes and protecting chondrocytes (Aldrich et al., 2023). Quercetin’s protective role in cartilage involves inhibiting MMPs and promoting autophagy, which helps maintain cartilage healthy (Li W. et al., 2021; Wang L. et al., 2022). However, like many natural compounds, quercetin has low bioavailability due to poor absorption and rapid metabolism (Carrillo-Martinez et al., 2024). Nanoparticle approaches, including quercetin-loaded liposomes, polymeric nanoparticles, solid lipid nanoparticles (SLNs), as while as nanostructured lipid carriers (NLCs) that are lipid-based delivery systems that combine both solid and liquid lipid, have been developed to enhance quercetin’s bioavailability, stability, and targeted delivery to inflamed joints, making it a more viable treatment option for OA (Carrillo-Martinez et al., 2024). Quercetin-loaded nanoparticles, when administered intra-articularly, showed prolonged retention in joint tissues, providing sustained release of quercetin and improving its anti-inflammatory effects in OA models (Akhileshwar Jha et al., 2024; Jennings et al., 2016). Besides the lipid nanoparticles, gold nanoparticles have been explored as carriers for various bioactive compounds, including quercetin. Due to their small size, AuNPs can efficiently deliver quercetin to targeted sites, enhancing cellular uptake and therapeutic efficacy (Sadalage et al., 2021).

##### 3.1.3.4 Baicalin (Scutellaria baicalensis)

Baicalin, a flavonoid derived from *Scutellaria baicalensis*, is known for its potent anti-inflammatory, antioxidant, and chondroprotective effects, making it an attractive candidate for the treatment of OA (Hu et al., 2022). However, like many other natural compounds, baicalin suffers from poor water solubility, low bioavailability, and rapid systemic clearance, which limit its therapeutic potential in clinical applications (Huang et al., 2019). To address these limitations, various nanoparticle-based delivery systems have emerged as a promising strategy to enhance effectiveness of baicalin in OA management.

Lipid-based nanoparticles, including liposome, SLNs and NLCs, have been explored for improving baicalin’s bioavailability and targeted delivery to inflamed joints (Zhang et al., 2016; Shi et al., 2016). There nanoparticles protect baicalin from rapid degradation, facilitate sustained release, and improve its solubility in biological fluids. For instance, baicalin-loaded SLNs have demonstrated enhanced anti-inflammatory activity and greater cartilage protection in OA models, compared to baicalin in its free form (Gao et al., 2022). Another innovative approach involves the use of polymeric nanoparticles, particularly those made from biodegradable materials like PLGA. These nanoparticles allow for the controlled release of Baicalin, ensuring prolonged therapeutic effects at the site of inflammation. Functionalization of these nanoparticles with targeting ligands, such as hyaluronic acid, further enhances their ability to accumulate in osteoarthritic joints by targeting CD44 receptors expressed on synovial cells, which are implicated in OA pathology (Gaio et al., 2020; Salathia et al., 2023; Bigaj-Józefowska and Grześkowiak, 2022).

Nanoparticles provide sustained release and enhanced targeting of inflamed tissues. Additionally, lipid-based nanoparticles, such as SLNs and nanoemulsions, can further improve baicilin’s solubility and systemic circulation, allowing for more efficient delivery to OA-affected joints (Ashfaq et al., 2023; Ghasemiyeh and Mohammadi-Samani, 2018). Moreover, nanofibers and hydrogels have been employed as localized delivery platforms for baicalin (Wang Z.-Z. et al., 2022; Liu et al., 2022c). These nanostructures can be injected directly into the joint space, providing a sustained release of baicalin over an extended period. This localized administration minimizes systemic side effects while maintaining high concentrations of the active compound in the affected joints (Bai et al., 2021).

##### 3.1.3.5 Andrographolide (AG)

Andrographolide (AG), extracted from *Andrographis panicula*, is a potent anti-inflammatory compound known for its ability to modulate immune responses and inhibit pro-inflammatory cytokine production (Xiong et al., 2021). Its application in OA treatment, however, is limited due to poor water solubility and rapid systemic clearance (Zhao et al., 2019). Recent advancements in nanotechnology have enabled the development of AG-loaded nanoparticles that can overcome these limitations. For instance, AG encapsulated in poly (acrylic acid)-modified mesoporous silica nanoparticles has been shown to provide a pH-responsive platform for sustained release, allowing the drug to be released more effectively in the acidic environments of inflamed OA joints (He et al., 2021). This targeted delivery not only improves AG’s therapeutic efficacy but also minimizes systemic side effects (Cheng et al., 2023). Another innovative approach involves the use of AG-loaded liposomes, which enhance the stability and bioavailability of AG, while allowing for controlled release in the affected joint tissues, thereby reducing inflammation and protecting cartilage from degradation. Although clinical applications of AG nanoparticles in OA remain in their early stages, preclinical studies suggest promising therapeutic outcomes (He et al., 2021; Hodgkinson et al., 2022).

##### 3.1.3.6 Diacerein (DIA)

Diacerein (DIA) is a widely studied anti-inflammatory drug used to slow the progression of OA (Bernetti et al., 2019). It inhibits the synthesis of pro-inflammatory cytokines like IL-1β, reducing cartilage degradation. However, DIA is associated with gastrointestinal side effects, which limit its long-term use (Panova and Jones, 2015). Nanoparticle-based delivery systems, such as PLGA nanoparticles, have been employed to enhance the bioavailability and minimize the adverse effects of DIA (Jung et al., 2020). DIA-loaded PLGA nanoparticles provide a sustained release profile, ensuring that therapeutic levels of the drug are maintained for extended periods within the joint space. This not only reduces the frequency of administration but also improves patient compliance (Jung et al., 2020). Another advanced approach involves the development of pH-responsive DIA-loaded nanoparticles, which allow for drug release specifically in inflamed environments, minimizing side effects while maximizing efficacy in OA treatment. Although human clinical trials are still ongoing, these innovations represent a significant step forward in enhancing the therapeutic utility of DIA for OA (Hu et al., 2020).

##### 3.1.3.7 Naringin nanoparticles

Naringin, a flavonoid derived from citrus fruits, has gained attention for its ability to promote cartilage regeneration and inhibit inflammatory pathways, making it a valuable candidate for OA therapy (Gan et al., 2023; Peng et al., 2024). However, like many other natural compounds, its clinical use is hindered by poor bioavailability and rapid clearance. Nanotechnology has provided solutions to these challenges through the development of advanced delivery systems. For example, polycaprolactone/polyethylene glycol-naringin (PCL/PEG-Nar) nanofiber membranes have been developed as a pH-responsive system for the sustained release of Naringin in OA-affected joints (Lan et al., 2020). This innovative approach ensures a steady release of the compound over time, reducing the severity of OA symptoms and promoting cartilage repair. Another promising technique involves the use of Naringin-loaded SLNs, which enhance the compound’s stability, bioavailability, and controlled release within joint tissues. SLNs have the added advantage of being biocompatible and biodegradable, making them an ideal platform for long-term OA treatment (Munir et al., 2021). While clinical applications of Naringin nanoparticles are still under investigation, preclinical studies have shown promising results, particularly in reducing inflammation and promoting cartilage regeneration in OA models (Ravetti et al., 2023).

### 3.2 Metallic nanoparticles in the treatment of OA

Oxidative stress, an imbalance between reactive oxygen species (ROS) and antioxidant defenses, plays a crucial role in the pathophysiology of OA. Excessive ROS disrupts redox signaling and damages key macromolecules such as proteins, lipids, and DNA, accelerating cartilage degradation and exacerbating joint inflammation (Ansari et al., 2020). In additionally, OA is also characterized by the activation of pro-inflammatory pathways, notably the NF-κB pathway, and the NLRP3 inflammasome, both of which contribute to the production of pro-inflammatory cytokines and enzymes responsible for cartilage breakdown (Ramirez-Perez et al., 2022; Xiao and Zhang, 2023; Yang et al., 2022).

Recent advances in nanotechnology have highlighted metallic nanoparticles (MNPs) as potential therapeutic agents for OA, not only as carriers for drugs and natural compounds but also as a promising therapeutic approach to directly counteract these destructive processes (Akhileshwar Jha et al., 2024). Metallic nanoparticles such as gold (AuNPs), silver (AgNPs), cerium oxide (CeO_2_NPs), and zinc oxide (ZnONPs) have shown potent antioxidant properties by scavenging ROS and reducing oxidative damage. Furthermore, these nanoparticles can directly modulate inflammatory processes and inhibit cartilage degradation, targeting key pathways like NF-κB and NLRP3 inflammasome activation, thus offering new possibility for OA treatment (Nayal et al., 2024).

#### 3.2.1 Gold nanoparticles

Gold nanoparticles (AuNPs) are extensively studied for their anti-inflammatory and antioxidative effects. AuNPs counteract oxidative stress, a major driver of OA, by scavenging ROS and restoring redox balance. Their inhibition of the NF-κB pathway reduces the production of pro-inflammatory cytokines like IL-1β and TNF-α, thus mitigating cartilage degradation and synovial inflammation (Wen et al., 2023; Abdel-Aziz et al., 2021). Functionalized AuNPs can neutralize ROS through interaction with protein thiol groups, further preventing cartilage damage. AuNPs also have favorable biocompatibility and low toxicity, though their tendency to accumulate raises concerns about long-term safety. Moreover, the high cost of gold limits large-scale production (Kus-Liśkiewicz et al., 2021).

#### 3.2.2 Silver nanoparticles

Silver nanoparticles (AgNPs) exhibit strong anti-inflammatory and antimicrobial properties, offering dual protection by reducing inflammation and preventing infections that may exacerbate OA (Ferdous and Nemmar, 2020; Gherasim et al., 2020). AgNPs effectively scavenge ROS and inhibit the NF-κB pathway, thereby lowering the production of matrix metalloproteinases (MMPs) and other cartilage-degrading enzymes (He et al., 2024; Akter et al., 2018). Furthermore, AgNPs can be incorporated into hydrogels or nanocomposites to provide sustained anti-inflammatory effects. However, their relatively higher cytotoxicity poses limitations for long-term use (Pangli et al., 2021; Nandhini et al., 2024).

#### 3.2.3 Cerium oxide nanoparticles

Cerium oxide nanoparticles (CeO_2_NPs), or nanoceria, stand out due to their redox-active properties, mimicking natural antioxidant enzymes like superoxide dismutase (SOD) and catalase (Dhall and Self, 2018). These nanoparticles continuously scavenge ROS through their ability to switch between Ce^3+^ and Ce^4+^ oxidation states, reducing oxidative stress in OA joints (Corsi et al., 2023; Xiong et al., 2023). CeO_2_NPs also inhibit NLRP3 inflammasome activation, which helps lower pro-inflammatory cytokines like IL-1β and IL-18 (Li et al., 2024a). Preclinical studies have shown that CeO_2_NPs preserve cartilage integrity and improve joint function, with low cytotoxicity making them suitable for long-term use (Xiong et al., 2023). However, the technical challenges in producing precise CeO_2_NPs and the need for further investigation into their long-term effects remain.

#### 3.2.4 Zinc oxide nanoparticles

Zinc oxide nanoparticles (ZnONPs) present a promising approach to OA treatment due to their antioxidant and anti-inflammatory effects (Lopez-Miranda et al., 2023). They modulate the NF-κB pathway, decreasing the production of pro-inflammatory mediators such as MMPs and cytokines, while directly scavenging ROS to prevent oxidative damage in cartilage and synovial tissues (Azeez et al., 2024). ZnONPs also enhance chondrocyte survival and promote cartilage regeneration by upregulating anabolic factors and increasing antioxidant enzyme production (Li et al., 2020; Mirza et al., 2015). Zinc’s affordability and the non-toxic byproducts of ZnONP degradation make them an appealing option for large-scale therapeutic applications, though more research is needed to ensure their long-term safety in clinical settings (Eker et al., 2024).

### 3.3 Molecular OA therapies enhanced by nanotechnologies

Nanotechnologies, when combined with gene-specific inhibitors like siRNA and shRNA, represent a promising frontier in OA therapy (Rai et al., 2019). Nanoparticles enhance drug delivery by increasing stability, targeting capabilities, and sustaining therapeutic release. This results in more precise modulation of key OA drivers, such as inflammation, oxidative stress, and cartilage degradation. Below, we explore the technical applications of various molecular therapies, such as Protein Kinase D (PKD) inhibitors, p47phox, p66shc, and the peptide KAFAK inhibitors in combination with the nanoparticle systems (Kumari et al., 2023; Li et al., 2024b; Yan et al., 2019; Jeong et al., 2020).

#### 3.3.1 Protein kinase D (PKD) inhibitors in nanoparticle systems

PKD is critical in regulating extracellular matrix (ECM) destruction and driving OA progression by activating the NF-κB pathway, which intensifies inflammation and matrix degradation (Baker et al., 2018). Nanoparticles, particularly PLGA, have been shown to deliver PKD inhibitors effectively, offering controlled and sustained release. This system enhances the inhibition of NF-κB activation and cytokine production (e.g., IL-1β), minimizing ECM degradation more effectively than free PKD inhibitors (Cho et al., 2019). Moreover, biomimetic nanoparticles, such as M2 macrophage-coated particles, exhibit enhanced targeting capabilities by mimicking immune responses, allowing for high concentrations of PKD inhibitors directly in inflamed joints, thereby reducing local inflammation. Biomimetic nanoparticles offer enhanced targeting of inflamed tissues, making them ideal for localized OA therapy (Cho et al., 2019; Rao and Shi, 2022). The choice of nanoparticle system should depend on the specific characteristics of the PKD inhibitor (e.g., hydrophobicity) and more clinical trials are needed for ideal therapeutic outcomes (e.g., sustained release, targeted delivery, or combination therapy) (Kumar et al., 2024; Tenchov et al., 2022; Yetisgin et al., 2020).

#### 3.3.2 Peptide KAFAK inhibitors

KAFAK is a peptide known for suppressing pro-inflammatory cytokines, including IL-1β and IL-6, key factors in OA pathology (Bartlett et al., 2013). Nanoparticles coated with M2 macrophage membranes, incorporating KAFAK, have demonstrated precision in targeting inflamed joints (Zhou et al., 2023). By modifying these nanoparticles with iRGD peptides and hyaluronic acid, sustained release is achieved, leading to reduced inflammation and protection of cartilage (Zhou et al., 2023; Zhang S. et al., 2022). The use of macrophage membrane coatings enhances precise delivery to inflamed joints and immune evasion, increasing the therapeutic efficacy of KAFAK inhibitors. However, the potential immune response to foreign coatings may necessitate further optimization through complex clinical trials (Zheng et al., 2023).

#### 3.3.3 shRNA-LEPR encapsulation in nanoparticle systems

The leptin receptor (LEPR) contributes to inflammation and cartilage breakdown in OA. Targeting LEPR with shRNA has proven effective in reducing its expression and mitigating inflammation. Biomimetic nanoparticles incorporating shRNA-LEPR, along with polyethylenimine (PEI) for gene delivery, offer sustained intra-articular release (Zhou et al., 2023; Li S. et al., 2024). Hyaluronic acid further enhances targeting to joint tissues, while M2 macrophage coatings improve localization in inflamed areas, efficient gene silencing on top of sustained release, leading to effective reduction in cartilage degradation (Li S. et al., 2024). However, long-term application maybe limited by the potential off-target effects, which could complicate the regulation of gene expression (Zha et al., 2021).

#### 3.3.4 siRNA-p47phox in PLGA nanoparticles

The p47phox subunit of NADPH oxidase is involved in reactive oxygen species (ROS) production, contributing to OA-related oxidative stress and cartilage damage. Encapsulating siRNA-p47phox in PLGA nanoparticles provides sustained release, reducing ROS production and inflammation (Shin et al., 2020a). This system not only alleviates oxidative damage but also preserves cartilage integrity for a prolonged therapeutic effect. The limited stability of siRNA in biological environments has hindered its clinical application in OA treatment (Kumari et al., 2023).

#### 3.3.5 siRNA-p66shc in nanoparticles

Overexpression of p66shc contributes to mitochondrial dysfunction and ROS overproduction in OA. Nanoparticles encapsulating siRNA-p66shc have shown efficacy in reducing ROS levels and inflammatory markers (e.g., IL-1β, TNF-α). By suppressing p66shc expression, these nanoparticles can modulate oxidative stress and inflammation, providing a targeted approach to slow OA progression (Shin et al., 2020b). Future studies should focus on optimizing the nanoparticle delivery efficiency and improving formulations stability (Liu et al., 2023a).

#### 3.3.6 P16INK4a and synovial inflammation

P16INK4a, a cell cycle regulator, is upregulated in fibroblast-like synoviocytes (FLS) during OA, contributing to joint damage (Damerau et al., 2024). siRNA targeting P16INK4a, encapsulated in PLGA nanoparticles, accumulates in synovial tissues and reduces inflammation by lowering IL-1β levels in FLS, providing a localized therapeutic option for specific reduction of synovial inflammation (Park et al., 2022). However, challenges such as off-target effects and delivery efficiency to specific joint compartments remain obstacles for clinical applications (Kumari et al., 2023; Li et al., 2024b).

#### 3.3.7 Black phosphorus nanosheets for cartilage and bone repair

Black phosphorus nanosheets represent a novel platform for OA treatment due to their pH-responsive behavior and ability to scavenge ROS (Zhang X. et al., 2022). These nanosheets promote cartilage regeneration and subchondral bone repair by protecting tissues from oxidative stress and modulating the joint’s inflammatory environment (Lu H. et al., 2023) (Table 1). However, research on black phosphorus nanosheet for OA treatment is still in the early-stages. Comprehensive clinical data are needed, particularly regarding sustained release, targeted delivery, combination therapies, and potential toxicity concerns (Zhuang et al., 2024).

**TABLE 1:** Nanomedicines to reduce inflammation of synovial and articular cartilage.

Composition	Cell	Animal	Dose	Outcome	Refernces
MLX-Ca (AC)_2_Lipo	ATDC5	Rats	*In vitro*: 20 μM *In vivo*:4 mM	Degenerated cartilage area↓	Hu et al., (2020)
CBD-PLGA-NPs	Rats’ primary chondrocytes	—	*In vitro*: 20 μg/mL *In vivo*:/	IL-1β, IL-6, TNF-α, MMP13↓	Gherasim et al., (2020)
SFNs-CXB	Human primary chondrocytes	—	*In vitro*: 800 μg/mL *In vivo*:/	ROS, IL-6↓	Pangli et al., (2021)
CUR-PLGA NPs	—	Rats	*In vitro*:/ *In vivo*: 200 mg/kg	NK-κB, Cleaved caspase3↓	Dhall and Self, (2018)
ACP	RAW264.7	Mice	*In vitro*: 10,25 μg/mL *In vivo*: 2.5, 5 mg/kg	TNF-α; IL-1β; ROS↓ Aggrecan↑ Degenerated cartilage area↓	Xiong et al., (2023)
PEG-FMN NPs	Rats’ primary chondrocytes	Rats	*In vitro*: 1.25 μg/mL, 14 μg/mL *In vivo*: 1.25 ug/mL	MMP13↓ Degenerated cartilage area↓	Azeez et al., (2024)
AG@MSNs-PAA	Rats’ primary chondrocytes	Rats	*In vitro*: 8 μM *In vivo*: 8 μM	MMP13↓ Aggrecan; COL2↑	Eker et al., (2024)
DIA-PLGA NPs	Rats’ primary synoviocytes	Rats	*In vitro*: 10 μg/mL *In vivo*: 90.61 μg/rat, 470.20 μg/mL	IL-1; IL-6; MMP3; COX-2; TNF-α↓ IL-4; IL-10↑	Li et al., (2024b)
MRC-PPL-PSO	Mice Primary chondrocytes	Mice	*In vitro*: 15 μM *In vivo*:15 μM	IL-1β; MMP3, TNF-α; MMP13; NF-κB, p-P38, p-AKT↓ COL2↑	Yan et al., (2019)
Ta-NH_2_ NPs	Rats’ primary chondrocytes	Rats	*In vitro*: 100 μg/mL *In vivo*: 10 μg	iNOS↓ Degenerated cartilage area, degenerated surface cartilage width, total osteophyte volume ↓	Bartlett et al., (2013)
AuNPs	—	Rats	*In vitro*:/ *In vivo*: AuNPs: 30 μg/kg	serum estrogen↓ IL-6, IL-β, TNF-α, COX-1, COX-2↓	Zhang et al., (2022b)
E@Au-Ag NPs	Rats’ primary chondrocytes	Rats	*In vitro*: 12 μg/mL *In vivo*:/	ROS↓ Apoptosis↓ Degenerated cartilage area↓	Zheng et al., (2023)
Au@PDA-WL NPs	ATDC5	Mice	*In vitro*: 15, 30, 60, 120, 240, 360 p.m. *In vivo*: 25 μL	Collage II↑ ROS↓ Degenerated cartilage area↓	Zha et al., (2021)
H-MnO_2_ NPs	—	Mice	*In vitro*:/ *In vivo*: 6 μg	IL-6; IL-1β; TNF-α↓ Degenerated cartilage area, degenerated surface cartilage width, total osteophyte volume ↓	Zhuang et al., (2024)
Mn_3_O_4_@CS	SW1353	Mice	*In vitro*: 8 μg/mL *In vivo*: 0.4 μg	iNOS, COX2, MMP13↓ SOD, CAT, COL2↑	Knights et al., (2023)
Mil-88a nano-enzyme	Mice Primary chondrocytes	Mice	*In vitro*:1–10 μg/mL *In vivo*:/	MMP13↓ SOD, Col2↑ Degenerated cartilage area, degenerated surface cartilage width, total osteophyte volume ↓	Van Osch et al., (2009)
PLGA NP AB	C28/I2	Mice	*In vitro*:10–60 μg/mL *In vivo*: 20 μg	Degenerated cartilage area, degenerated surface cartilage width, total osteophyte volume ↓	Jeyaraman et al., (2024)
PLGA-HA	RAW264.7	Mice	*In vitro*:3.9, 7.8, 15.6, 31.25, 62.5, 125 μg/mL *In vivo*: 10 mg/mL	NO↓	Roseti et al., (2019)
HA-NP	Mice primary chondrocytes	Mice	*In vitro*: 80 μg/mL *In vivo*: 0.2 mg/mL	NK-κB, MMP3, MMP13,COX-2, PGE_2_↓ Degenerated cartilage area↓	Liang et al., (2023)
PEG-PLGA-HA	C28/I2	Mice	*In vivo*: 2.3 mg/mL	Degenerated cartilage area, degenerated surface cartilage width, total osteophyte volume ↓	Wang et al., (2024)
PLEL@PL-NPs	ATDC5; primary human articular chondrocytes	Rats	*In vitro*: 0–2,500 μg/mL *In vivo*: 50 μL	IL-6, TNF-α, iNOS, COX-2,CD68↓ COL2↑ Degenerated cartilage area↓	Wu et al., (2024)
PDKi-NPs	Pigs’ primary chondrocytes	—	*In vitro*: 10 μM *In vivo*:/	Caspase3, p-Akt, NO, PGE2, NF-κB, MMP14, P53↓ ACAN, COL2, SOX9↑	Chen et al., (2020)
macrophage membrane-coated KAFAK-shRNA-LEPR-PEI-NPs	RAW264.7	Rats	*In vitro*: 1 μg/mL *In vivo*:/	TNF-α; IL-2β, CD86↓ IL-10, COL2, CD2↑	Atwal et al., (2023)
P47phox siRNA-PLGA-NPs	—	Rats	*In vitro*:/ *In vivo*: 0.2 μM	ROS↓ Degenerated cartilage area↓	Carton and Malatesta, (2024)
P16INK4a siRNA-PLGA-NPs	Primary cultured human articular chondrocytes and fibroblast-like synoviocytes	Mice	*In vitro*: 50, 100, 200,600, 1,000 μg/mL *In vivo*: 200 μg	TNF-α, IL-1β, IL-6, MMP13↓	Li et al., (2024d)
BPNSs	Rats’ primary chondrocytes	Rats	*In vitro*: 10 μg/mL, 20 μg/mL *In vivo*: 10 μg/mL	ADAMTS5, ADAMTS1↓ COL2, Aggrecan, RUNX2, BMP2↑	Jiang et al., (2024)

SFNs: silk fibroin nanoparticles; CXB: celecoxib; ACP: acid-activatable curcumin polymer; PEG: poly (ethylene glycol); FMN: formononetin; AG: andrographolide; MSNs: mesoporous silica nanoparticles; PAA: pH-responsive polyacrylic acid; DIA: diacerein; PLGA: poly (d,l-lactide-co-glycolide); NPs: nanoparticles; MRC-PPL: cartilage-targeting and OA-specific theranostic nanoplatforms; PSO: psoralen; Au: Gold; DIA: diacerein; Ta-NH_2_-NPs: tantalum nanoparticles; E@Au-Ag NPs: EGCG (Epigallocatechin gallate) decorated Au-Ag nano-jars; PDA: polydopamine; WL: WYRGRL; H-MnO_2_: Hollow- MnO2; HA: hyaluronic acid; PLEL: poly (d, L-lactide)-poly (ethylene glycol)-poly (d, L-lactide); PL: platelet lysate; PDKi: protein kinase D inhibitor. BPNSs: Black phosphorus nanosheets; CS: chondroitin sulfate.

In summary, nanoparticle-based delivery systems for molecular OA treatments offer numerous advantages, including enhanced targeting, sustained release, and reduced side effects. However, challenges such as off-target effects, nanoparticle stability, and scaling up for clinical applications remain. Future studies should prioritize optimizing nanoparticle formulations, enhancing bioavailability, and conducing long-term safety evaluations.

## 4 Nanomedicines for cartilage regeneration in OA

While inflammation plays a significant role in the progression of OA, regenerating damaged cartilage is key to achieving long-term disease modification (Knights et al., 2023). The unique challenges posed by cartilage, such as its avascularity and limited cellular repair mechanisms, make effective regeneration difficult (Van Osch et al., 2009; Jeyaraman et al., 2024; Roseti et al., 2019). Nanotechnology offers a promising avenue for overcoming these obstacles, providing innovative strategies that focus on cartilage repair and regeneration. In this chapter, we explore advanced nanomedicine approaches that aim to restore cartilage integrity, utilizing nanoparticles, scaffolds, and biologically active molecules for sustained, localized, and effective treatment (Eftekhari et al., 2020; Liang et al., 2023).

### 4.1 Key nanomedicines that promote cartilage regeneration

While inflammation is a significant contributor to OA progression, the ability to regenerate damaged cartilage remains a critical challenge in achieving meaningful disease modification (Roseti et al., 2019). Current pharmacological and surgical interventions often fail to fully restore articular cartilage due to the tissue’s limited self-repair capabilities. The avascular nature of cartilage, combined with its low density of chondrocytes, restricts its ability to recover from injury, leading to the gradual deterioration of joint function (Wang et al., 2024).

Traditional regenerative approaches, such as cell-based therapies and growth factor injections, have shown potential but are hampered by issues such as poor cell survival, lack of integration with native tissues, and the inability to control the precise delivery of therapeutic agents over time (Tsujii et al., 2024). These limitations highlight the need for advanced strategies that can enhance cartilage repair while ensuring sustained, localized effects (Wang et al., 2024).

Nanotechnology offers transformative solutions to these challenges by enabling the controlled and targeted delivery of bioactive molecules directly to sites of cartilage damage (Qiao et al., 2022). Nanomedicines can be engineered to deliver a variety of therapeutic agents—including growth factors, cytokines, and gene therapies—in a sustained manner, maximizing their efficacy (Liu et al., 2023b). Nanofibrous scaffolds, designed to mimic the extracellular matrix (ECM) of cartilage, provide structural support for cell attachment and proliferation. These scaffolds can be functionalized with growth factors or cells to further promote cartilage regeneration (Huang et al., 2024).

Key growth factors, such as transforming growth factor-beta (TGF-β) and bone morphogenetic proteins (BMPs), play pivotal roles in cartilage repair (Zhang X. et al., 2022; Wu et al., 2024). However, delivering these proteins effectively is challenging due to their short half-life and rapid degradation in the joint environment (Thielen et al., 2019). Nanoparticle carriers, such as PLGA nanoparticles combined with nanofibrous scaffolds, have demonstrated success in encapsulating these growth factors, providing sustained release while protecting them from degradation. These integrated nanotechnologies have been shown to stimulate chondrocyte activity more effectively, thereby enhancing cartilage regeneration (Chen et al., 2020; Qin et al., 2023).

Additionally, injectable hydrogels containing nanoparticles and cartilage-promoting factors can fill cartilage defects, offering both mechanical support and bioactivity. These hydrogels are often modified with materials like hyaluronic acid or collagen to better mimic the native cartilage environment. As they promote the growth of new cartilage tissue, they also integrate with existing tissues, with their biodegradability ensuring gradual replacement by natural tissue (Atwal et al., 2023; Carton and Malatesta, 2024).

### 4.2 Exosome-mimicking nanoparticles

Exosome-mimicking nanoparticles represent an innovative therapeutic strategy for OA by replicating the biological functions of natural exosomes, which are small cell-derived vesicles involved in intercellular communication and tissue repair (Chavda et al., 2023). These engineered nanoparticles hold promise in drug delivery, gene therapy, and tissue regeneration, offering targeted solutions for modulating the inflammatory and degenerative processes associated with OA (Li L. et al., 2024).

Natural exosomes, typically 30–150 nm in diameter, are secreted by various cell types such as mesenchymal stem cells (MSCs) and chondrocytes. They facilitate the transfer of bioactive molecules, including proteins, lipids, and RNA, which play critical roles in inflammation modulation and cartilage repair (Jiang et al., 2024). MSC-derived exosomes can promote chondrocyte migration and proliferation via the Mir-106b-5P/TIMP2 signaling pathway or activating YAP through the Wnt pathway (Tao et al., 2017; Tan et al., 2020). They can also reduce MMP13 and ADAMTS5 levels in chondrocytes, reverse mitochondrial membrane potential changes, and alleviate OA (Cosenza et al., 2017), potentially by inhibiting phosphorylation of p38 and ERK and promoting protein kinase B phosphorylation (Zhai et al., 2022). However, their clinical application faces challenges, such as low yield, heterogeneity, and instability during storage. Exosome-mimicking nanoparticles address these limitations by offering greater stability, scalability, and the ability to customize properties for specific therapeutic needs (Wang X. et al., 2022; Tan et al., 2024).

These nanoparticles are engineered to emulate the structural and functional characteristics of natural exosomes. Constructed from biodegradable materials like liposomes, polymeric nanoparticles, or silica nanoparticles, they are functionalized with surface proteins, ligands, or targeting molecules like hyaluronic acid or MSC-derived membrane proteins. These surface modifications enable the delivery of bioactive molecules, such as miRNAs or proteins, to stimulate cartilage regeneration without the complexity of direct stem cell therapies (Li Y.-J. et al., 2021). Several studies have shown the potential of exosome-mimicking nanoparticles in OA treatment. For instance, nanoparticles mimicking exosomes derived from bone marrow mesenchymal stem cells (BM-MSCs), loaded with growth factors and RNA molecules, have been shown to reduce cartilage degradation, improve joint function, and lower pro-inflammatory cytokine levels in OA models (Cosenza et al., 2017). Additionally, HA-coated exosome-mimicking nanoparticles have demonstrated effective delivery of siRNA targeting MMP13—an enzyme involved in cartilage breakdown—leading to enhanced cartilage preservation in animal studies (Kumari et al., 2023; Qiu et al., 2013) (Table 2).

**TABLE 2:** Nanomedicine that promotes cartilage regeneration and repair.

Composition	Cell	Animal	Dose	Outcome	References
KGN-PLGA-PEG-PLGA-BMSCs	—	Rabbits	*In vitro*:/ *In vivo*: 50 mg/kg	Degenerated cartilage area↓	Zhou et al. (2015)
CD90@ NPs	—	Rabbits	*In vitro*:/ *In vivo*:12 mg/rat	CD68, iNOS↓ IL-10, IGF1, CYCLIN B↑ Degenerated cartilage area, degenerated surface cartilage width, total osteophyte volume ↓	Cosenza et al. (2017)
hASC-EVs	Human chondrocytes-osteoarthritis	Rats	*In vitro*: 1×10^8^, 2×10^8^ particles/mL *In vivo*: 1×10^8^ particles/rat	MMP1, MMP3, MMP13, ADAMTS5, IL-1β, CD86↓	Zhai et al. (2022)
PPD-MSC-sEVs	Human chondrocytes-osteoarthritis	Mice	*In vitro*:/ *In vivo*: 1×10^7^ particles/mice	ADAMTS5, MMP13, IL-1β, TNF-α↓ Aggrecan, COL1↑	Li et al. (2021a)
MMP13 siRNA NPs	ATDC5	Mice	*In vitro*:/ *In vivo*: 1,875 nmol	MMP13↓ Degenerated cartilage area, degenerated surface cartilage width, total osteophyte volume ↓	Kalashnikova et al. (2020)

KGN: kartogenin; BMSCs: bone marrow MSCs; mPEG-Hz-b-PCL: methoxy poly (ethylene oxide)-hydrazone-poly (ε-caprolactone) copolymers; CD90@ NPs: CD90^+^ MCS-derived micro-vesicle-coated nanoparticle; hASC-EVs: human adipose-derived stem cells extracellular vesicles; PPD: ε-polylysine-polyethylene-distearyl phosphatidylethanolamine; MMP: matrix metalloproteinase.

### 4.3 Mesenchymal stem cells in nanoparticle therapy

Nanoparticle-encapsulated mesenchymal stem cell (MSC) therapy is a promising new approach for OA treatment, combining the regenerative capabilities of MSCs with the advantages of nanoparticles, such as targeted delivery and enhanced stability (Ding et al., 2022; Bunnell, 2021; Mizuno et al., 2022; Hoang et al., 2022). This method addresses key limitations of traditional MSC-based therapies, such as low cell survival, poor retention in joint tissues, and insufficient targeting (Zou et al., 2023). By using nanoparticles, the therapeutic potential of MSCs in cartilage repair, inflammation modulation, and slowing OA progression is significantly enhanced (Cai et al., 2023).

**FIGURE 3:**
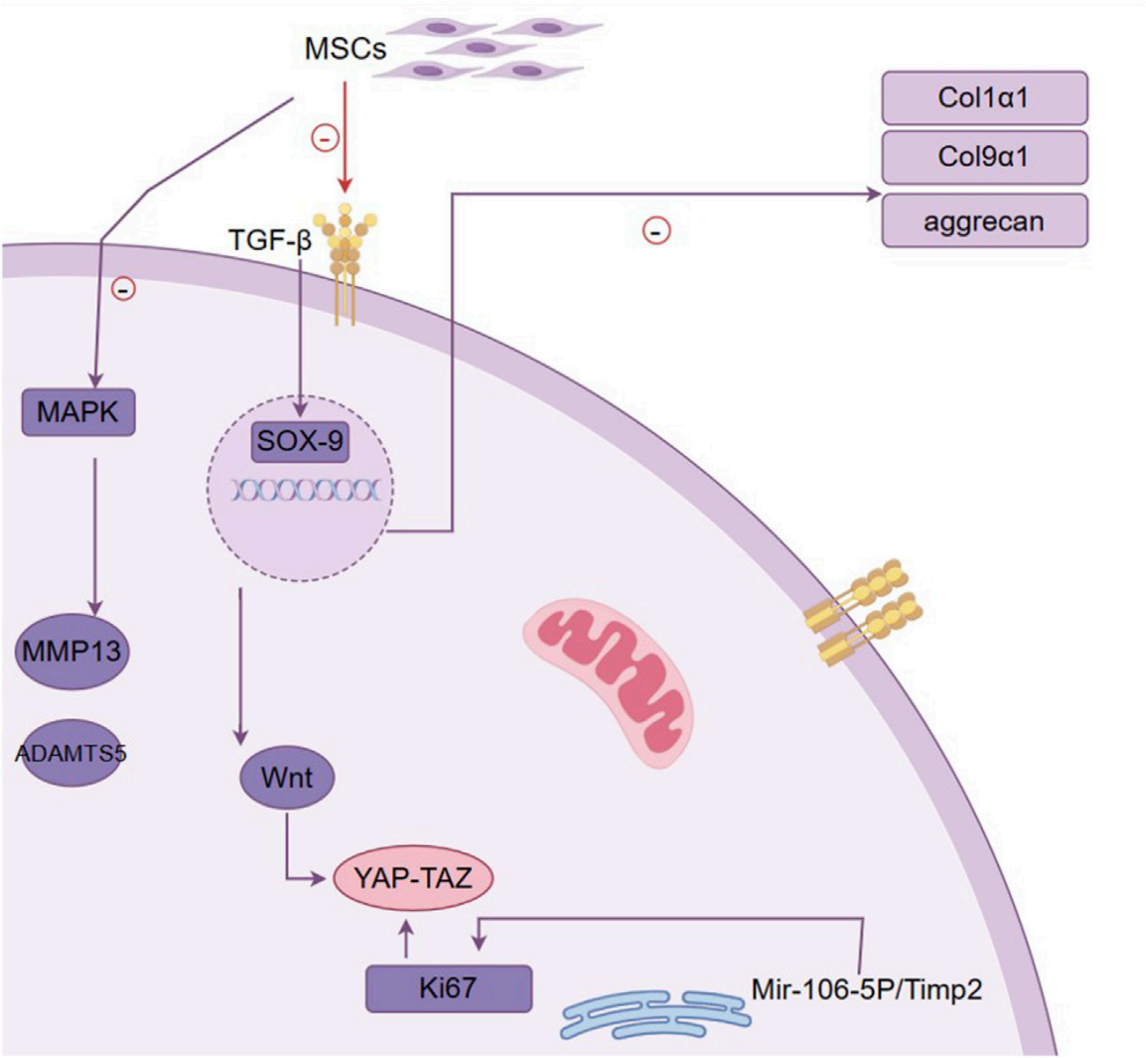
Mechanism of MSC in promoting cartilage repair. This figure outlines the pivotal role of mesenchymal stem cells (MSCs) in cartilage repair and the molecular mechanisms involved. It demonstrates how MSCs contribute to the restoration and maintenance of cartilage through their differentiation into chondrocytes, influenced by signaling molecules such as TGF-β. The figure highlights the activation of the transcription factor SOX-9, which is essential for chondrogenic differentiation and is upregulated by TGF-β. SOX-9 then promotes the expression of key extracellular matrix (ECM) components, including type II collagen (Col2a1) and aggrecan, which are crucial for cartilage structure and function. Additionally, the figure indicates the involvement of the MAPK pathway, which can lead to increased expression of MMP13 and ADAMTS5, enzymes associated with cartilage degradation. The interaction between the Wnt pathway and YAP-TAZ signaling is also depicted, illustrating their role in cell proliferation and tissue regeneration. The figure encapsulates the complex network of interactions that underlie MSC-induced chondrogenesis, highlighting the therapeutic potential of MSCs in treating osteoarthritis by enhancing cartilage repair and reducing inflammation.

MSCs are multipotent cells that can differentiate into chondrocytes, the cells responsible for maintaining cartilage integrity. This chondrogenic potential is linked to the upregulation of SOX9, a key marker for chondrocyte progenitors (Zhao et al., 2017). MSCs also secrete bioactive molecules like TGF-β and BMP, which promote bone formation and increase extracellular matrix (ECM) production by inducing SOX9 expression (Cai et al., 2023; Du et al., 2023). Studies have shown that MSC injections can restore chondrocyte proliferation, inhibit apoptosis, regulate inflammation, and help ki67 expression in damaged cartilage (Zhang et al., 2021) (Figure 3). However, despite these benefits, MSCs often exhibit poor retention and rapid degradation after intra-articular injection, limiting their therapeutic efficacy.

To overcome these challenges, various types of nanoparticles are being investigated to encapsulate MSCs or their secreted products. These include polymeric nanoparticles, liposomes, and hydrogels. For example, PLGA nanoparticles have been used to encapsulate MSCs or MSC-derived exosomes, promoting chondrocyte proliferation and enhancing cartilage matrix production. In preclinical models, this approach has shown potential in reducing cartilage degradation (Fan et al., 2006). Nanoparticles can also deliver anti-inflammatory cytokines, such as IL-10 and TGF-β, in a controlled manner, leading to reduced synovitis and cartilage destruction. While most research in nanoparticle-encapsulated MSC therapy remains at the preclinical stage, early results in animal models are promising (Gonzalez-Fernandez et al., 2022). These studies demonstrate improved cartilage regeneration, reduced inflammation, and better joint function. However, further investigation is required to optimize nanoparticle formulations, ensure long-term safety, and assess the clinical efficacy of these therapies in human OA patients (Seo et al., 2022).

## 5 Conclusion: Recent advances in nanotechnologies for OA treatment

Recent advancements in nanotechnologies offer promising avenues for overcoming the limitations of traditional OA therapies. Nanoparticles have been utilized as vectors to improve the delivery, bioavailability, and stability of existing OA treatments, such as NSAIDs, by enabling targeted, sustained release directly into the inflamed joints. Metal-based nanoparticles, particularly those leveraging silver and copper, have shown potential in reducing oxidative stress, though concerns about toxicity and cost remain.

The combination of nanoparticles with molecular agents, including peptides, siRNA, and shRNA, further enhances the therapeutic potential by allowing for precise targeting of inflammation, oxidative stress, and cartilage degradation at the molecular level. Additionally, nanomedicines designed to promote cartilage regeneration, such as black phosphorus nanosheets, represent a breakthrough in the tissue repair process. Another emerging area is the encapsulation of mesenchymal stem cells (MSCs) in nanoparticles, which improves the efficacy of stem cell therapies in repairing joint tissues and reducing inflammation.

While these nanotechnologies demonstrate significant promise, their clinical applications are still in early stages, with most studies focusing on animal models. Continued research is essential to address challenges related to nanoparticle safety, long-term effects, and scaling up for clinical use. Future directions will likely involve refining nanoparticle formulations for better bioavailability, stability, and minimizing off-target effects, ensuring that nanomedicine becomes a transformative tool in OA management.
